# Application of the oblique lateral interbody fusion technique in salvage surgery: technical note and case series

**DOI:** 10.3389/fsurg.2023.1144699

**Published:** 2023-05-19

**Authors:** Jialuo Han, Shuo Han, Shengwei Meng, Xiaodan Zhao, Hao Zhang, Jianwei Guo, Derong Xu, Houchen Liu, Mingrui Chen, Xuexiao Ma, Yan Wang

**Affiliations:** ^1^ Department of Spine Surgery, The Affiliated Hospital of Qingdao University, Shandong, China; ^2^ Department of Radiology, The Affiliated Hospital of Qingdao University, Shandong, China

**Keywords:** failed posterior interbody fusion surgery, adjacent vertebral disease, salvage surgery, oblique lateral interbody fusion, OLIF51, OLIF25, technical note

## Abstract

**Objective:**

The oblique lateral interbody fusion (OLIF) technique is a promising interbody fusion technique. This study summarizes the technical aspects of OLIF as a salvage surgery and the preliminary outcomes of a series of cases.

**Patients and methods:**

A retrospective review of patients with leg or back pain induced by pseudoarthrosis or adjacent segment disease after posterior lumbar interbody fusion/transforaminal lumbar interbody fusion was done. These patients underwent salvage OLIF surgeries in our institution from January 2021 to March 2022. Variables such as the demographic, clinical, surgical, and radiological characteristics of the enrolled patients were recorded and analyzed.

**Results:**

Eight patients (five females and three males; mean age 69.1 ± 5.7 years, range 63–80 years) were enrolled in this study. The mean operative time was 286.25 min (range: 230–440 min), and the estimated blood loss was 90 ml (range: 50–150 ml). Only one of the eight patients experienced a complication of lower limb motor weakness, which disappeared within 5 days after surgery. The latest data showed that the mean intervertebral space height increased from 8.36 mm preoperatively to 12.70 mm and the mean segmentary lordosis increased from 8.92° preoperatively to 15.05°. Bone fusion was achieved in all but one patient, who was followed up for only 3 months. The JOA scores Japanese Orthopaedic Association (JOA) Scores for low back pain of all patients significantly improved at the final follow-up.

**Conclusion:**

OLIF provides a safe and effective salvage strategy for patients with failed posterior intervertebral fusion surgery. Patients effectively recovered intervertebral and foraminal height with no additional posterior direct decompression.

## Introduction

Spinal diseases have become the leading cause of global labor losses because of their high disability rate, slow recovery rate, and youth-targeted characteristics (1). Lumbar interbody fusion (LIF) is a classic and effective procedure for treating degenerative, infectious, traumatic, and neoplastic lumbar diseases. Over the decades, a variety of surgical approaches have been developed to achieve LIF, among which posterior lumbar interbody fusion (PLIF) and transforaminal lumbar interbody fusion (TLIF) have been used as representatives until now (2). With their wide application, the number of patients experiencing failed posterior intervertebral fusion surgery, such as internal fixation fracture, intervertebral space infection, and cage migration, is increasing. In addition, fixation and fusion lead to biomechanical changes, and there is also an alarming increase in the occurrence of adjacent segment disease (ASD). If the patient experiences a recurrence of severe symptoms after primary surgery, salvage surgery should be performed immediately (3). However, the loss of the posterior bone-ligament structure, the extensive scar hyperplasia, and the adhesion of the dural and neural tissues make it extremely difficult to deal with the intervertebral space, and even a small surgical error will lead to irreparable and serious consequences, which poses great challenges and is difficult to revise.

The lateral lumbar interbody fusion (LLIF) technique, which includes oblique lateral interbody fusion (OLIF) and extreme lateral interbody fusion (XLIF), was developed to gain lateral access to the intervertebral space by bypassing the posterior structures, to achieve indirect decompression and interbody fusion (4). Among them, the recently developed OLIF, which is proving beneficial because of its retroperitoneal physiological space approach, dispenses with the need to divide the psoas major muscle, which has attracted the attention of surgeons (5). Therefore, OLIF may be an excellent method to perform salvage surgery in patients with failed posterior fusion surgery.

At present, there are a few reports on the application of OLIF in salvage surgery. In particular, different technical specifications are recommended for the treatment of failures of different segments (L2-5 or L5/S1) and characters during the performance of OLIF. In this study, we systematically summarize the technical notes of the OLIF salvage procedure and analyze the outcomes of the case series for a professional reference.

## Materials and methods

This study retrospectively reviewed patients who underwent salvage OLIF surgery at our institution from January 2021 to March 2022. The final decision on the surgical strategy to be adopted was agreed upon by the medical team, which included anesthesiologists and spine surgeons. All surgeries were performed by the same experienced physician. The procedures are described in detail in the next section. The demographic, clinical, surgical, and radiological characteristics of the enrolled patients were recorded and analyzed, mainly including the following variables: sex, age, type of lesion, segment, JOA score, JOA recovery rate, follow-up period, operative time, estimated blood loss, segmental lordosis, disc space height, and intervertebral fusion. All data were obtained through the extraction of inpatient medical records and regular follow-ups. The study was approved by the Ethics Committee (approval number: QYFY WZLL 27203) of the Affiliated Hospital of Qingdao University, and informed consent was obtained from the patients.

## Surgical technique

**Figure 1:**
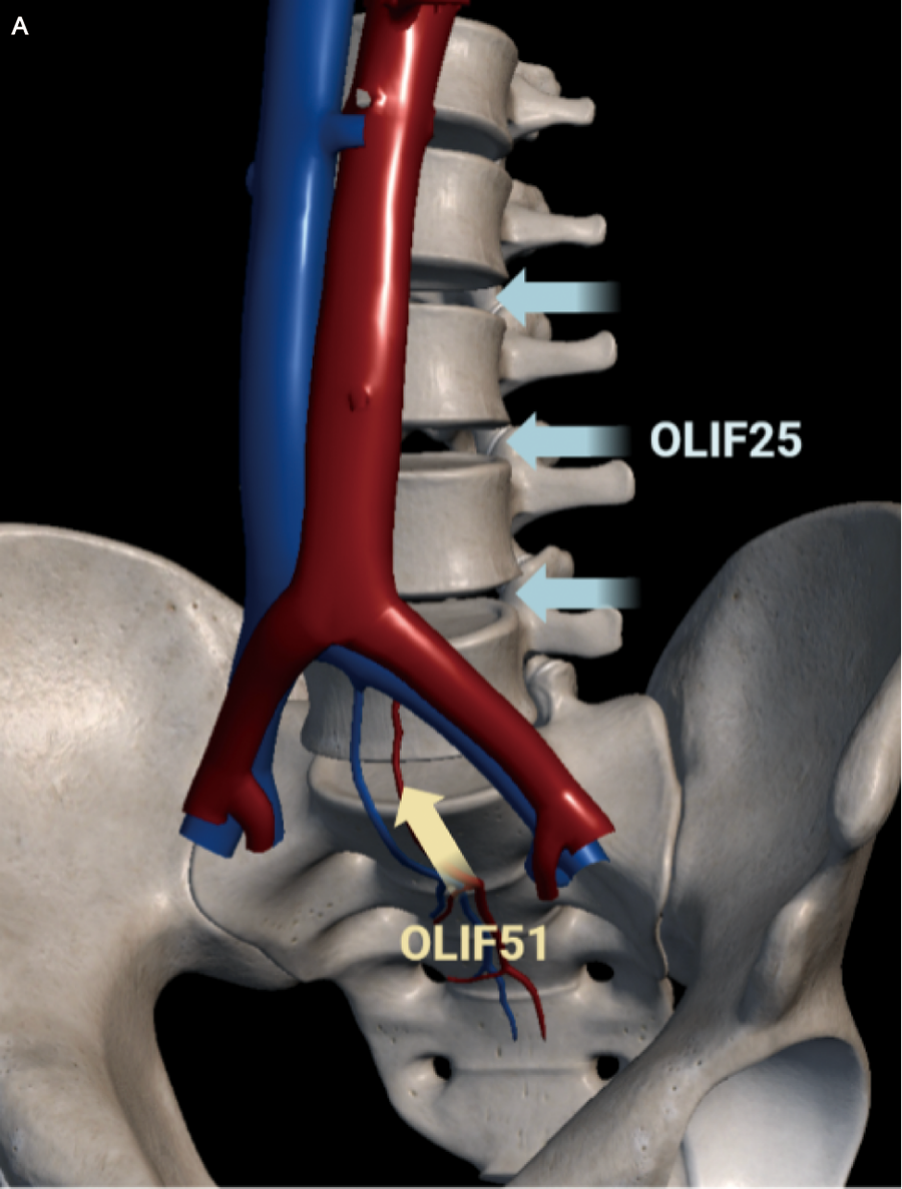
Schematic of a surgical window for OLIF2/5 and OLIF5/1.

Salvage OLIF surgeries were divided into OLIF25 and OLIF51, corresponding to L2-5 and L5/S1 intervertebral discs, respectively. The main difference between these is that the surgical window of the former is located between the psoas major and the abdominal aorta, while the latter is located at the bifurcation point of the iliac vessels (Figure 1).

### Preoperative assessment

Anatomical evaluation of the patient before surgery is necessary to ensure a smooth procedure and avoid vascular and nerve damage.

First, it must be determined whether the patient has had any prior surgery involving the retroperitoneum, as retroperitoneal tissue adhesion will seriously hinder the performance of the surgical procedure. Here, emphasis must be placed on the evaluation of the anatomy of the anterior great vessels through MRI and enhanced 3D CT, which will help eliminate surgical contraindications and allow the provision of a choice of specific procedures. Ideally, the left common iliac vessel will emerge from the L5 vertebral portion without straddling the L4/5 or L5/S1 intervertebral spaces, allowing for a comfortable OLIF25 or OLIF51. If the left common iliac vessel originates at the level of the target intervertebral space, the procedure will cause excessive interference, and the surgeon should carefully consider whether OLIF is a reasonable option in this situation.

The pelvis should also be considered when performing this procedure. The high iliac crest affects OLIF25 at the L4/5 level but does not significantly influence OLIF51. Conversely, the relative position of the symphysis pubis to the parallel line of the L5/S1 intervertebral space may hinder surgery in the L5/S1 intervertebral space.

### Anesthesia and position

The surgery was performed under general anesthesia. When performing revision surgery, it is generally necessary to make a position switch between the right lateral decubitus and the prone decubitus, and the surgeon should choose the first position from the perspective of the case characteristics, surgical strategy, and lumbar stability. It is important that the coronal plane of the body, whether the right lateral decubitus or the prone decubitus, lies perpendicular or parallel to the floor to provide the best reference axis for perspective and manipulation. The OLIF51 procedure slightly differs from that of OLIF25 in that it requires the left leg to be extended, otherwise, it becomes difficult to expose the incision site. Pelvic fixation can be performed by using an oblique fixation band, ignoring the pubic symphysis to avoid covering the surgical instruments and incisions.

### Incision design

**Figure 2:**
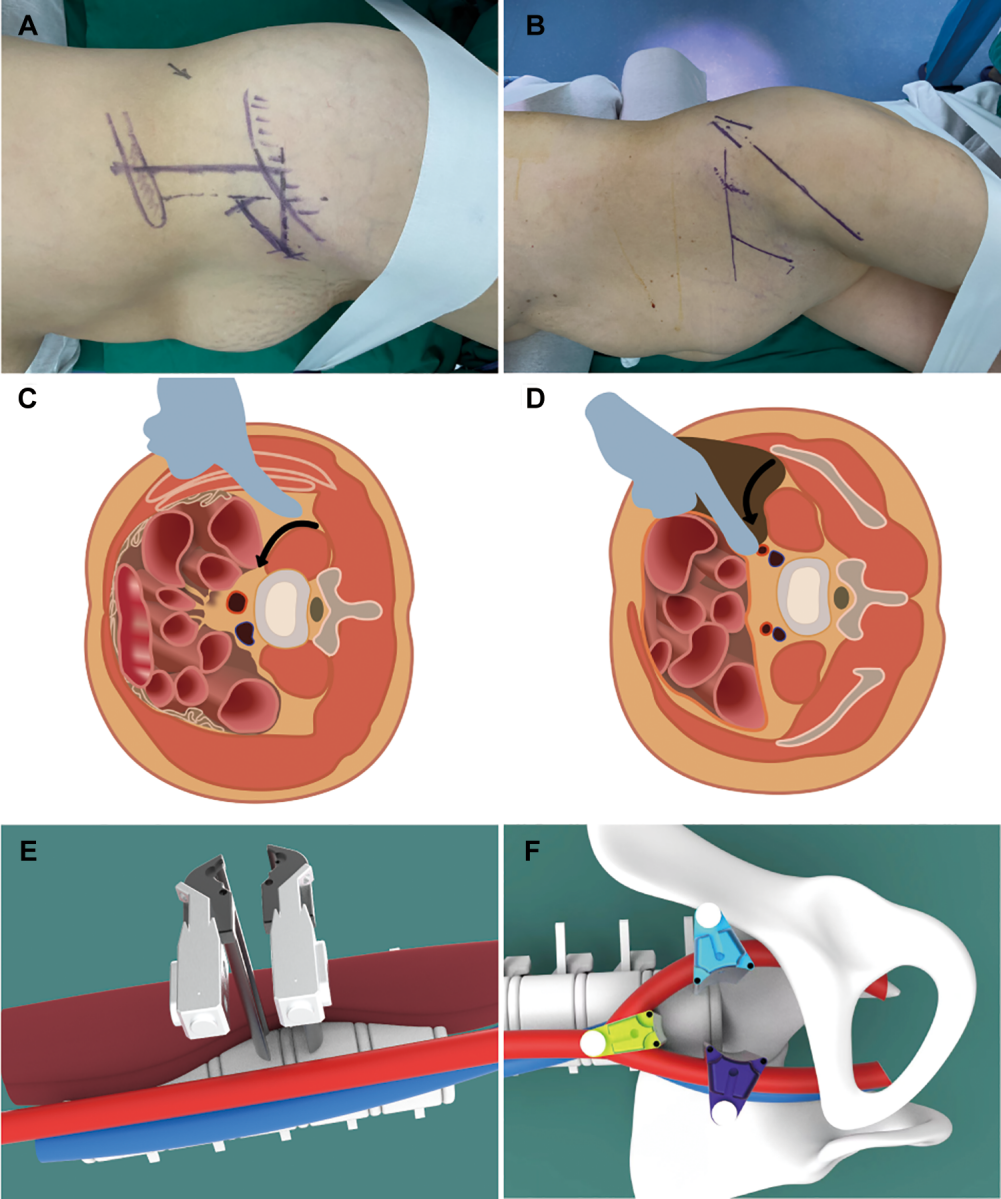
Schematic diagram of an OLIF2/5 surgical incision design, separation approach, and pull hook placement (**A, C, E**). Schematic diagram of an OLIF5/1 surgical incision design, separation approach, and pull hook placement (**B, D, F**).

Before designing the incision in OLIF25, the 12th rib and the iliac crest were outlined on the skin to ensure that they would not cover the incision. Then, the surface projection of the target intervertebral space was marked, and its center point was determined under vertical fluoroscopy. An incision parallel to the anterior superior iliac crest was made 4–5 cm anterior to the central point, which was approximately 4–7 cm in length (Figure 2A).

The incision in OLIF51 was done more ventrally to provide a more oblique surgical approach to the bifurcation of the iliac vessels. The lateral body surface projection of the iliac crest and the L5/S1 intervertebral space were marked by fluoroscopy. From the central point of the target disc, two identical lines were drawn: a vertical line projected perpendicular to the floor, and another one extending on the abdomen in the direction of the disc. A 5 cm incision should be made along the pelvis at two fingers’ breadth from the anterior superior iliac spine. In this case, a longer and more ventrally incision was made because of the excessive pelvic incidence angle (Figure 2B).

### Retroperitoneal approach for disc repair

A blunt dissection of the abdominal external oblique, internal oblique, transversalis muscle, and transversalis fascia was performed. After exposing the retroperitoneal fat, a circumferential sweep was performed below the transversalis fascia with the fingers parallel to the abdominal wall to first separate the posterior peritoneum. The separation was then continued posteriorly along the inner abdominal wall or the inner pelvis until the psoas major muscle was palpated.

The next exposure procedure was slightly different between the two techniques. OLIF25 was advanced along the anterior margin of the psoas to the anterior disc space. The retractor was placed after the anterior disc space was widened by pushing the psoas and the pulsating abdominal aorta beneath it laterally (Figures 2C–E). For OLIF51, the pulsating common iliac artery and its deep common iliac vein were first palpated along the anterior margin of the psoas. The retractor was placed at the anterior disc space, which was exposed by blunt dissection of the bifurcated iliac vessels (Figures 2D–F). The entire procedure should be performed with maximum direct visualization.

### Preparation of the intervertebral space

After the ideal exposure of the target disc, the annulus fibrosus was cut close to the endplate to prepare the intervertebral space. The cage was loosened using Cobb’s torque-limiting shaft and removed with a clamp. If necessary, this step can be completed by using a power tool, such as a high-speed drill. Then, a curette and reamer were used to thoroughly clean the scar tissue in the intervertebral space and expose the endplate, taking care not to induce additional endplate injury. The intervertebral space was sequentially distracted by using different-sized trials until a satisfactory intervertebral height was obtained. An appropriate cage (10–20 mm, 6°–24°) loaded with an autogenous or allogenic bone was implanted into the intervertebral space.

The differences between the OLIF25 technology and the OLIF51 technology mainly lie in the annulotomy position and surgery direction. The former was placed more lateral to the disc and the cage was implanted parallel to the coronal plane of the body, while the latter was placed on the ventral side of the disc and the cage was implanted in a sagittal plane parallel to the body. In practice, frequent perspective positioning is necessary because the surgeon is prone to disorientation.

## Results

### Patients’ demographics

We identified eight patients who underwent salvage OLIF surgery. Table 1 summarizes the patients’ baseline demographics. Among the eight subjects, there were five women and three men, with a mean age of 69 years. The types of lesions that led to revision surgery in these patients included adjacent vertebral disease (three patients), pseudoarthrosis (two patients), cage protrusion (three patients), and an internal fixation fracture (one patient). The surgical levels were L3/4 in two patients, L4/5 in four patients, L5/S1 in one patient, and L2/4 in one patient.

**Table 1:** Demographic data.

Case No.	Gender/Age	Lesion type	Segment	Primary diagnosis	Primary surgery	Symptom
1	F/63	Pseudarthrosis + cage protrusion	L4/5	Scoliosis + spinal stenosis	TLIF	Low back pain + leg pain
2	M/64	Fixation fracture + pseudarthrosis	L2–4	Vertebral infection	Total *en bloc* spondylectomy	Low back pain
3	M/64	Cage protrusion	L3/4	Spondylolisthesis + spinal stenosis	TLIF	Low back pain + leg pain
4	F/68	ASD	L4/5	Spondylolisthesis	PLIF	Low back pain + leg pain
5	F/69	ASD	L4/5	Spinal stenosis	PLIF	Low back pain + leg pain
6	F/72	Fixation loosening + cage protrusion	L5/S1	Spondylolisthesis + spinal stenosis	TLIF	Low back pain + leg pain
7	F/73	ASD	L3/4	Spinal stenosis	PLIF	Low back pain
8	M/80	Cage protrusion	L4/5	Spinal stenosis	PLIF	Low back pain

M, male patient; F, female patient; ASD, adjacent segment disease; TLIF, transforaminal lumbar interbody fusion; PLIF, posterior lumbar interbody fusion; pseudoarthrosis, more than 3 mm of translational motion or more than 5° of angular motion on flexion and extension radiographs.

### Surgical data and clinical outcomes

Perioperative data and clinical outcomes are summarized in Table 2. The mean follow-up was 9 months (range: 6–12 m), mean operative time was 286.25 min (range: 230–440 min), and the estimated blood loss was 90 ml (range: 50–150 ml). The mean JOA score improved significantly from 11.37 (range: 8–14) preoperatively to 21.87 (range: 18–26) at the last follow-up. Only one of the eight patients experienced a complication of lower limb motor weakness, which resolved within 5 days of surgery.

**Table 2:** Surgical data and clinical outcomes.

Case No.	Salvage surgery	Estimated blood loss (ml)	Operative time (min)	Perioperative complications	Follow-up (m)	JOA score for low back pain (max: 29)		
						Preop	Postop (recovery rate)	
1	OLIF 25	150	230	—	8	8		18 (47.6%)
2	OLIF 25	100	300	—	3	13		22 (56.2%)
3	OLIF 25	70	240	—	12	12		21 (52.9%)
4	OLIF 25	50	270	—	12	14		24 (66.7%)
5	OLIF 25	50	270	—	6	14		26 (80%)
6	OLIF 51	100	440	—	8	10		22 (63%)
7	OLIF 25	150	280	Lower extremities motor weakness	8	11		21 (56%)
8	OLIF 25	50	260	—	6	9		21 (60%)
Mean ± SD		90 ± 42.42	286.25 ± 65.89		7.87 ± 3.04	11.37 ± 2.26		21.87 ± 2.36

Recovery rate = [postoperative score − baseline score]/[29 − baseline score] × 100 (%).

### Radiographic evaluation

Standing lumbar spine radiographs and lumber CT were the regular follow-up re-examinations. The most recent data revealed that the mean intervertebral space height increased from 8.36 mm preoperatively to 12.70 mm, and the mean segmental lordosis increased from 7.05° preoperatively to 13.30°. Bone fusion was achieved in all but one patient, who was followed up for only 3 months (Table 3).

**Table 3:** Radiographic results.

Case No.	Segmental lordosis (°)		Disc space height (mm)		Interbody fusion
	Preoperative	Postoperative	Preoperative	Postoperative	
1	9.6	14.3	5.42	9.56	Y
2	−5.2	11.2	15.08	18.96	N
3	9.6	11.8	7.93	10.94	Y
4	10.6	12.5	7.75	11.57	Y
5	13.2	16.3	5.95	12.93	Y
6	15.8	23.3	8.37	13.27	Y
7	11.8	16.5	10.13	13.65	Y
8	6	14.5	6.24	10.73	Y
Mean ± SD	8.92 ± 6.39	15.05 ± 3.86	8.36 ± 3.11	12.7 ± 2.89	
*P*	0.036		0.012		

## Illustrative cases

### Case 1

**Figure 3:**
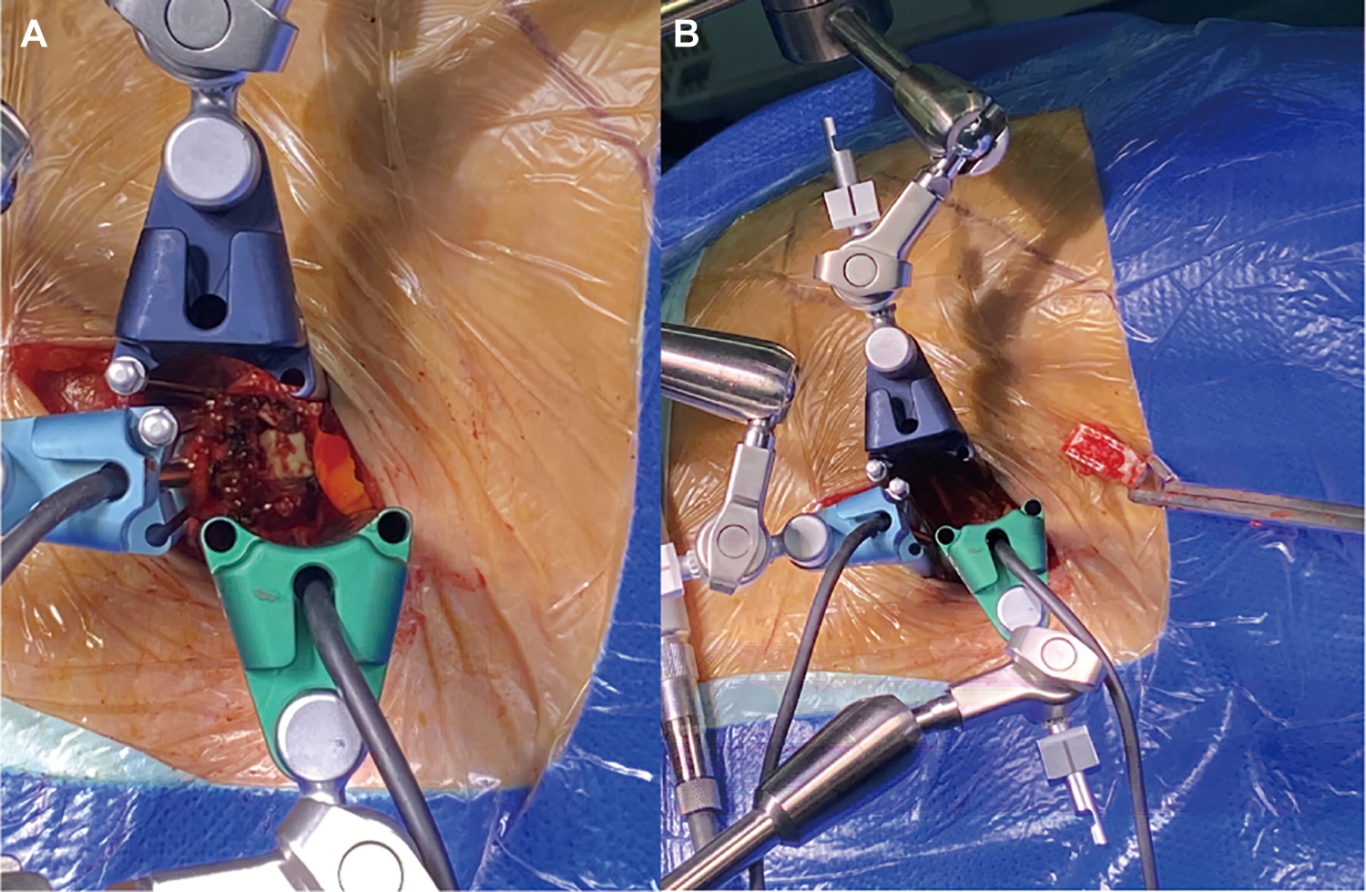
Exposure of a displaced cage from an oblique anterior view (**A**). Successful removal of the displaced cage was (**B**).

**Figure 4:**
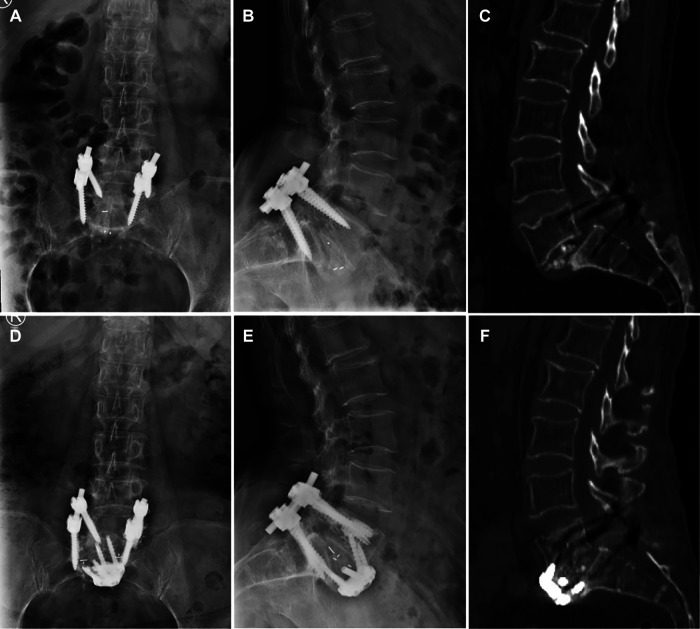
Preoperative frontal and lateral x-rays and CT, screw extraction, anterior slippage of a vertebral body, and cage displacement (**A–C**). Satisfactory repositioning of the vertebral body at 6 months postoperatively (**D, E**), with bone bridging seen on CT (**F**).

A 72-year-old woman underwent an L5/S1 TLIF for low back pain and neurogenic claudication caused by L5/S1 spondylolisthesis with secondary spinal stenosis. Symptoms resolved postoperatively until 5 months after surgery, when the patient complained of recurrent low back pain and radicular pain in the right lower extremity. A radiologic assessment revealed that the L5 pedicle screw had become loose and the cage at L5/S1 had migrated beyond the anterior edge of the S1 vertebral body. In addition, dual X-ray absorptiometry indicated the presence of severe osteoporosis (*T* = −3.7). Given the requirements of removing the migration cage in the presence of posterior scar adhesions and providing strong fixation in this case, we performed anterior removal of the cage and OLIF51 and replaced the loose screws with cement-augmentation of pedicle screws after confirming the accessibility of the oblique corridor (Figure 3). The procedure was successful, with no postoperative complications. Follow-up imaging 6 months after the revision surgery showed that the L5/S1 intervertebral space had formed a bony fusion and the patient’s chief complaint was fully resolved (Figure 4).

### Case 2

**Figure 5:**
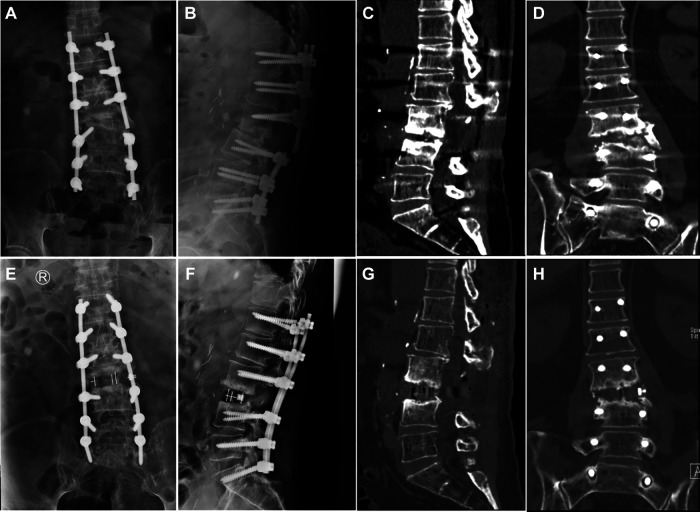
Preoperative frontal and lateral x-rays and CT showed bone resorption with bilateral rod fractures and pseudarthrosis formation (**A–D**). Postoperative images show a satisfactory reconstruction of lumbar curvature and height (**E–G**). A restored coronal balance (**H**).

A 64-year-old man underwent vertebral excision, intervertebral bone grafting, and posterior fixation for vertebral infection following L3 percutaneous vertebroplasty. However, the patient experienced severe low back pain for 3 years after surgery. Plain radiographs and CT images showed bone resorption in the spaces between L2 and L4 with vertebral endplate osteosclerosis with bilateral rod fractures, and pseudoarthrosis formation at L2/4. After careful consideration, we decided to use the OLIF25 technique to thoroughly clean the vertebral space and implant a wide OLIF cage (6°, 14 mm × 55 mm) to restore its height through an anterolateral approach. Then, the posterior program was to simply replace the fracture-connecting rod. Postoperative imaging confirmed that the revision surgery successfully restored lumbar curvature and height, and the patient achieved great relief from low back pain (Figure 5).

## Discussion

Failed posterior fusion surgery is not uncommon in the clinic, for which timely and appropriate salvage surgery is recommended (6). Here, we report a series of cases of patients in whom the salvage OLIF technique was applied to treat failed posterior intervertebral fusion surgery, and initial follow-up data demonstrated that the protocol was effective.

The most common reasons for failed posterior interbody fusion are endplate injury, inadequate space opening, inappropriate cage shape, small cage size, inadequate depth of cage implantation, poor cage fit to the bony endplate of the spinal canal, and inadequate internal fixation strength. These are also always accompanied by many unfavorable factors, such as, for example, advanced age and severe osteoporosis (7–9). Unfortunately, posterior reinvasion is extremely challenging and risky. The loss of the posterior bone-ligament structure makes dissection and exposure difficult, and scar formations and adhesion of the dural and neural tissues greatly increase the risk of bleeding and nerve injury during dissection (10, 11). Moreover, it is difficult to obtain a sufficient surgical field to remove the original cage and clear the intervertebral space by pulling the dural sac and nerve root. In addition, removal of the cage during salvage surgery sometimes results in endplate destruction and even fractures, making it difficult to ensure effective intervertebral height restoration and fusion by reinsertion of a small cage.

In 1997, Mayer reported the technique of lumbar interbody fusion from the space between the abdominal vascular sheath and the anterior border of the psoas muscle, which provided a new perspective for the approach to lumbar fusion (12). In 2012, Silvestre improved and developed the OLIF-specific cage and corresponding channel system based on the original approach, thus representing the OLIF technique and concept, which was rapidly popularized and applied worldwide (4). The OLIF procedure helps the surgeon approach the target disc through the physiologic space between the retroperitoneal abdominal vascular sheath and the anterior border of the psoas muscle, with no damage to the posterior structures. At the same time, the oblique lateral corridor provides a wide window for intervertebral space manipulation, which makes it possible to utilize a large cage bridge for bridging the bilateral edge of the apophyseal ring, thus meeting the demand for revision surgery to restore intervertebral height and achieve rigid fusion. Previous reports suggest that the OLIF technique is suitable for the treatment of various kinds of lumbar diseases, as it restores intervertebral stability and intervertebral space height, achieves indirect decompression, and corrects the lumbar sequence (13). Therefore, it is reasonable to believe that it will also help avoid revision surgery after a failed posterior intervertebral fusion surgery.

Phan and Mobbs reported a case of salvage OLIF for non-union following posterior surgery at the L2/3 level, demonstrating the acceptable OLIF approach to achieve satisfactory interbody fusion (14). In the study by Orita et al., a follow-up based on the JOA score demonstrated the high clinical efficacy of salvage OLIF in the treatment of failed spinal surgery with a mean recovery rate of 65.0% and more effective interbody fusion (15). In addition, compared with the traditional posterior revision surgery, salvage OLIF technology also helped to achieve effective control of blood loss, which was also demonstrated in our study (mean blood loss of 90 ml). In our study, the illustrative cases of patients were more complex because their condition was accompanied by severe intervertebral height loss or osteoporosis. Due to technical and instrumental deficiencies, it may be difficult to achieve satisfactory efficacy for this condition by using the posterior approach. However, our preliminary follow-up results show that the oblique lateral approach, the large OLIF cage, and the complementary anterior fixation can perfectly solve the aforementioned problems. The most common complications of OLIF surgery reported in the literature are vascular, ureteral, and nerve damage, in addition to lower-extremity weakness. In our case series, only one patient developed postoperative lower-extremity weakness but recovered quickly. We suggest that the above complications can be effectively avoided by performing blunt dissection with maximum visualization.

The potential shortcomings of OLIF as a salvage surgery are as follows: there was no significant increase in the complication rates of OLIF as a salvage surgery, but the presence of postoperative scarring or adhesions around the spinal root may reduce the indirect decompressive effect (16). This may result in poor clinical outcomes for salvage surgery compared to primary surgery.

This study had several limitations. First, this was a single-center retrospective study with a small sample size. Second, the follow-up period was short, and no control group was established. Therefore, more prospective, controlled studies with large samples are necessary to overcome these drawbacks.

## Conclusions

OLIF can effectively restore intervertebral and foraminal height in patients with failed posterior interbody fusion surgery with no additional posterior direct decompression. A lower risk of bleeding and nerve damage gives OLIF great potential and application prospects in the field of lumbar salvage surgery.

## Data Availability

The original contributions presented in the study are included in the article, further inquiries can be directed to the corresponding authors.
